# A Randomized Controlled Trial of Emotion Regulation Therapy for Psychologically Distressed Caregivers of Cancer Patients

**DOI:** 10.1093/jncics/pkz074

**Published:** 2019-09-13

**Authors:** Mia S O’Toole, Douglas S Mennin, Allison Applebaum, Britta Weber, Hanne Rose, David M Fresco, Robert Zachariae

**Affiliations:** 1 Unit for Psychooncology and Health Psychology, Aarhus University and Aarhus University Hospital, Denmark; 2 Department of Psychology, Teachers College, Columbia University, New York, NY; 3 Department of Psychiatry & Behavioral Sciences, Memorial Sloan Kettering Cancer Center, New York, NY; 4 Department of Oncology, Aarhus University Hospital, Denmark; 5 Department of Psychological Sciences, Kent State University, Kent, OH

## Abstract

**Background:**

Previous cognitive behavioral therapies for informal caregivers (ICs) have produced negligible effects. The purpose of this study was to evaluate, in a randomized controlled trial, the efficacy of Emotion Regulation Therapy adapted for caregivers (ERT-C) on psychological and inflammatory outcomes in psychologically distressed ICs and the cancer patients cared for.

**Methods:**

A total of 81 ICs with elevated psychological distress were randomly assigned to ERT-C or a waitlist condition and assessed pre-, mid-, and post-treatment. In 52 cases, the patient cared for by the IC was included. Patients did not receive ERT-C. Both the ERT-C and waitlist groups were followed 3 and 6 months post-treatment. Data were analyzed with multilevel models, and *P* values were two-sided.

**Results:**

Compared with ICs in the waitlist condition, ICs in the ERT-C condition experienced medium to large statistically significant reductions in psychological distress (Hedge’s *g *=* *0.86, 95% confidence interval [CI] = 0.40 to 1.32, *P <* .001), worry (*g *=* *0.96, 95% CI = 0.50 to 1.42, *P <* .001), and caregiver burden (*g *=* *0.53, 95% CI = 0.10 to 1.99, *P* = .007) post-treatment. No statistically significant effects were found for rumination (*g *=* *0.24, 95% CI = −0.20 to 0.68, *P* = .220). Results concerning caregiver burden were maintained through 6 months follow-up. Although the effects on psychological distress and worry diminished, their end-point effects remained medium to large. No statistically significant effects on systemic inflammation were detected (C-reactive protein: *g* = .17, 95% CI = −0.27 to 0.61, *P* = .570; interleukin-6: *g* = .35, 95% CI = −0.09 to 0.79, *P* = .205; tumor necrosis factor-alpha: *g* = .11, 95% CI = −0.33 to 0.55, *P* = .686). Patients whose ICs attended ERT-C experienced a large increase in quality of life post-treatment (*g *=* *0.88, 95% CI = 0.18 to 1.58, *P* = .017).

**Conclusions:**

To our knowledge, this is the first randomized controlled trial evaluating the efficacy of ERT-C for ICs. Given the previous disappointing effects of other cognitive behavioral therapies for this population, the present findings are very encouraging. Identifying ICs with elevated psychological distress and providing them with relevant psychotherapy appears an important element of comprehensive cancer care.

Cancer rates are increasing globally (1), resulting in more patients as well as informal caregivers (ICs) involved in their care. Meta-analyses reveal elevated levels of psychological distress expressed as anxiety and depression in ICs (2,3), which may even exceed the levels experienced by patients (2,4,5) and often persists into their loved one’s survivorship (6). ICs also experience health complications, including sleep difficulties and fatigue (7,8) and cardiovascular disease (9), leading to increased mortality (10).

Our recent meta-analysis found that cognitive behavioral therapies (CBTs) produce negligible effects (Hedge’s *g* = 0.04) (11,12), with minimal effect sizes obtained (*g *<* *0.2) for all seperate types of outcomes (ie, psychological, interpersonal, and physical well-being). One important reason for this disappointing effect may be the way in which IC distress has been conceptualized and approached (12,13). Recent studies have pointed to the presence of perseverative negative thinking among ICs (eg, 14,15). Perseverative negative thinking refers to verbal, self-referential mental processes, such as worry and rumination, oftentimes employed as a means to diminish negative emotional experiences. However, these strategies are more likely to worsen and prolong rather than alleviate psychological distress (16,17). Three studies in the meta-analysis were mindfulness-based interventions, which could be argued to more directly offer the client training in alternatives to perseverative negative thinking than traditional CBTs (eg, 18,19). Only one of these studies employed a randomized design, and more research is needed to evaluate the efficacy of such approaches within an IC context. 

Emotion Regulation Therapy (ERT) belongs to the CBT family but was explicitly developed to target perseverative negative thinking by cultivating healthier emotion regulation skills supported by mindfulness practices. ERT was originally developed to treat chronic anxiety with or without depression and has demonstrated clinical efficacy (20–22). We recently adapted ERT for the caregiver population (ERT-C) and reported initial efficacy in an open-label, single-arm trial (23), including reduced depression and anxiety symptoms and perseverative negative thinking (*g-*range = 0.36–0.92), although not caregiver burden (*g *=* *0.15). Although promising, this trial design cannot account for spontaneous improvement over time and did employ follow-up assessments.

Investigating the effect of psychotherapy beyond self-report measures of psychological distress is important. Individuals with distress, including ICs, may experience increased systemic inflammatory biomarkers (eg, C-reactive protein [CRP]) (24–26), on which we demonstrated a reduction following psychotherapy in a recent meta-analysis (27). Finally, there is evidence to suggest that reducing the distress of ICs through psychotherapy has a buffering effect on patient well-being (28,29) and thus should be investigated in relation to ERT-C.

This study sought to build on our promising findings. The primary purpose of this study was to evaluate, in a randomized controlled trial, the efficacy of ERT-C on psychological and inflammatory outcomes in psychologically distressed ICs and the cancer patients cared for. We hypothesized that 1) ERT-C would demonstrate an advantage compared with a waitlist condition concerning primary (ie, caregiver burden, worry, rumination, and psychological distress) and secondary outcomes (ie, emotion regulation, quality of life, sleep quality, and pro-inflammatory markers), 2) the effect would be maintained through follow-up, and 3) patient outcomes (quality of life, psychological distress, and inflammation) would improve acutely and be maintained through follow-up.

## Methods

The trial was pre-registered at Clinicaltrials.gov (#NCT02322905), and the study protocol was approved by the local ethics committee (#1–10-72–430-14). Reporting of trial procedures and results follow the CONSORT guidelines (30).

### Participants

ICs were recruited from August 2015 until December 2017 through Aarhus University Hospital Oncology Department teams treating lung, gastrointestinal, and gynecological cancers. Originally, only ICs of lung and gastrointestinal cancers were considered for inclusion. Due to a slower-than-expected inclusion rate and internal reorganization of the oncology departments, other cancer types were also considered. ICs could have any relationship with the patient (eg, friend, spouse) but had to self-identify as being a caregiver of the patient. Inclusion criteria were: >4 on the Distress Thermometer (31); elevated perseverative negative thinking (ie, Brooding Subscale of the Rumination Response Scale [RRS-B] >12) or worry (Brief Penn State Worry Questionnaire [PSWQ] >15) (32,33); and a remaining lifetime expectancy of the patient of >6 months. ICs were eligible for inclusion despite patients declining participation, and multiple ICs of the same patient could participate. Exclusion criteria were: an expected survival of the patient of <6 months; active substance abuse; and participation in other psychosocial trials. Based on a power calculation (α = 0.0125; 1-β = 0.90), a dropout rate of 35%, and an intraclass correlation between ICs of the same patient of 0.20, the inclusion of 80 ICs in total would yield a statistically significant acute treatment effect of a medium effect size (Cohen’s *d *=* *0.5) (11) when evaluated with a 2 (group; ERT-C vs waitlist) × 3 (time; pre, mid, post) mixed linear model (MLM). Due to multiple primary outcomes for ICs, the alpha-level was reduced to 0.0125 (0.05÷4) for those.

Using PASS software (34), a randomization list was generated by the last author (RZ), who did not have any contact with participants. To conceal the result of the final ticket, the number of participants in each group was not fully balanced (±10%).

### Procedure

Upon oral consent from the ICs, they were screened with the Distress Thermometer at the oncology department. If meeting the criterion, both IC and patient were informed in more detail about the study. The patient provided written consent for study participation at the oncology department, and the IC provided written consent to be contacted by phone regarding further screening for eligibility. In case the IC also met cutoff scores for perseverative negative thinking, an assessment meeting was scheduled. Upon completing baseline questionnaires, ICs were allocated to either ERT-C or a waitlist condition. Outcome questionnaires were completed at baseline, mid-treatment (4 weeks), and post-treatment (8 weeks). Inflammatory biomarkers were obtained from blood samples at pre- and post-treatment. Upon completing the 8-week waitlist period, IC controls were offered ERT-C. Follow-up assessments were completed at 3 and 6 months post-treatment. All questionnaires were completed in person and administered via paper.

### Conditions

ERT-C is a manualized treatment consisting of eight weekly sessions (35). The first half (sessions 1–4) focuses on psychoeducation and training of emotion regulation skills, including attentional regulation skills (ie, shifting and sustaining attention on a difficult experience) and metacognitive regulation skills (ie, decentering from and reappraisal of emotional experiences), with four different mindfulness practices. In the second half (sessions 5–8), clients are encouraged to deploy regulation skills during exposure towards the pursuit of personally meaningful activities, which are simultaneously perceived as rewarding (eg, spending time with a friend) and threatening (eg, worrying about leaving sick husband alone). The presence of both reward and threat is termed a motivational conflict and serves as the context for exposure exercises with the aim of obtaining a more motivationally balanced approach to one’s life (36).

All sessions had a duration of 60 minutes and took place at the local university. Twelve master’s or doctorate-level students delivered the treatment. They were all trained in ERT-C and received weekly face-to-face supervision with prior review of audio-recorded sessions by the first author (MSO’T) as well as monthly supervision from the second author (DSM). To establish adherence to the treatment protocol, a primary coder coded all eight sessions from 25% of cases, with 40% of these cases coded by a secondary coder. The coders were research assistants trained in the principles of ERT-C but had not been involved in the treatment. Based on session audio recordings, coders rated the presence or absence of 72 manual components. Therapists on average addressed 85% of the components, and the intraclass correlation between the two coders was .77. The waitlist condition also spanned 8 weeks.

### Materials

#### Screening Materials

The Distress Thermometer (31) was used to screen for psychological distress. Perseverative Negative Thinking was measured with the five-item RRS-B (32) and five items from the PSWQ (37), validated as a brief version (33).

#### Primary outcomes for ICs

Caregiver burden was assessed by the Caregiver Reaction Assessment (CRA) (38), measuring multiple dimensions of caregiver burden, including self-esteem, family support, finances, schedule, and health (α = .58). Higher scores indicate higher burden.

Psychological distress was evaluated using the Hospital Anxiety and Depression Scale (HADS; α = .85) (39). Higher scores indicate higher levels of distress.

Perseverative negative thinking was measured with the full version of the PSWQ (α = .84) and the brooding subscale of the RRS-B (α = .79). Higher scores on both measures indicate higher levels of perseverative negative thinking.

#### Secondary outcomes for ICs

Quality of life in ICs was measured with the World Health Organization (WHO)-5 questionnaire (40) (α = .90). Higher scores indicate better quality of life. Sleep quality was measured with the Pittsburgh Sleep Quality Index (41). Higher scores indicate poorer sleep quality. Emotional closeness was assessed with the discrepancy between two items, rated on a scale from 0 to 10, namely, actual and ideal emotional closeness (42). Negative scores indicate less emotional closeness than wanted.

Pro-inflammatory markers included high sensitivity-CRP, tumor necrosis factor-alpha (TNFα), interleukin (IL)-1, and IL-6. To control for diurnal variation, samples were obtained within ±3 hours of each other. Serum was analyzed with the high-sensitivity CRP enzyme-linked immunosorbent assay kit and the V-PLEX Proinflammatory Panel 1 (Human) kit, Meso Scale Discovery, Rockville, MD. Higher scores indicate higher levels of inflammation.

#### Model-related outcomes for ICs

Emotion regulation was assessed using four measures. The Difficulties in Emotion Regulation Scale (43) measures difficulties with aspects of emotion regulation (α = .94). Higher scores indicate more difficulties with emotion regulation. The Five Facet Mindfulness Questionnaire (44) measures elements of mindfulness (α = .81). Higher scores indicate higher levels of mindfulness. Decentering was assessed with the Experiences Questionnaire (45) (α = .93). Higher scores indicate greater decentering abilities. Cognitive reappraisal was assessed with the reappraisal subscale of the Emotion Regulation Questionnaire (46) (α = .90). Higher scores indicate more use of cognitive reappraisal.

#### Patient outcomes

Quality of life was the primary outcome for patients, evaluated with the European Organization for Research and Treatment of Cancer Core Quality of Life Questionnaire (QLQ-C30) (47) global quality of life (α = .90). Psychological distress was evaluated using the HADS (39). Proinflammatory markers (hs-CRP, TNFα, IL-1, IL-6) were evaluated, but it was not possible to control for diurnal variation in patients.

### Analytic Strategy

All inflammatory outcomes were log-transformed, and outliers (>3 SDs) were deleted (0–2 scores on each outcome). The acute effect of ERT for ICs was explored with a 2 (group; ERT-C vs waitlist) × 3 (time; pre, mid, post) MLM, where time at level 1 was nested within participants at level 2. Three-level MLMs, with patients at level 3, were evaluated but did not converge, possibly due to low variation at the third level. The two groups were combined to determine the effect through the follow-up period. MLMs were employed to evaluate the best fit of time, including a linear effect (continued improvement), a log-linear effect (maintenance of effect), and a quadratic effect (worsening). To compare endpoint effects between measures, a linear function of time was estimated from pre-treatment through follow-up.

All MLMs were based on the intent-to-treat sample (cf 48). Intercepts were specified as random in all models and so was the slope if it improved the model fit (ie, −2LL change). Missing data at the item level were handled by mean substitution, which was only considered for participants with less than 50% missing data on a particular scale (cf 49). Results revealed that missing data at the item level were minimal and below 2% for any of the primary outcomes. Cohen’s *d* was derived from the F-test calculated as *d *=* *2 × √(F/df) and transformed into Hedges’ *g* (50). An effect size of 0.2, 0.5, and 0.8 was considered small, medium, and large, respectively (11). *P* values were two-sided. All MLMs were performed in SPSS-25. 

## Results

**Figure 1.**
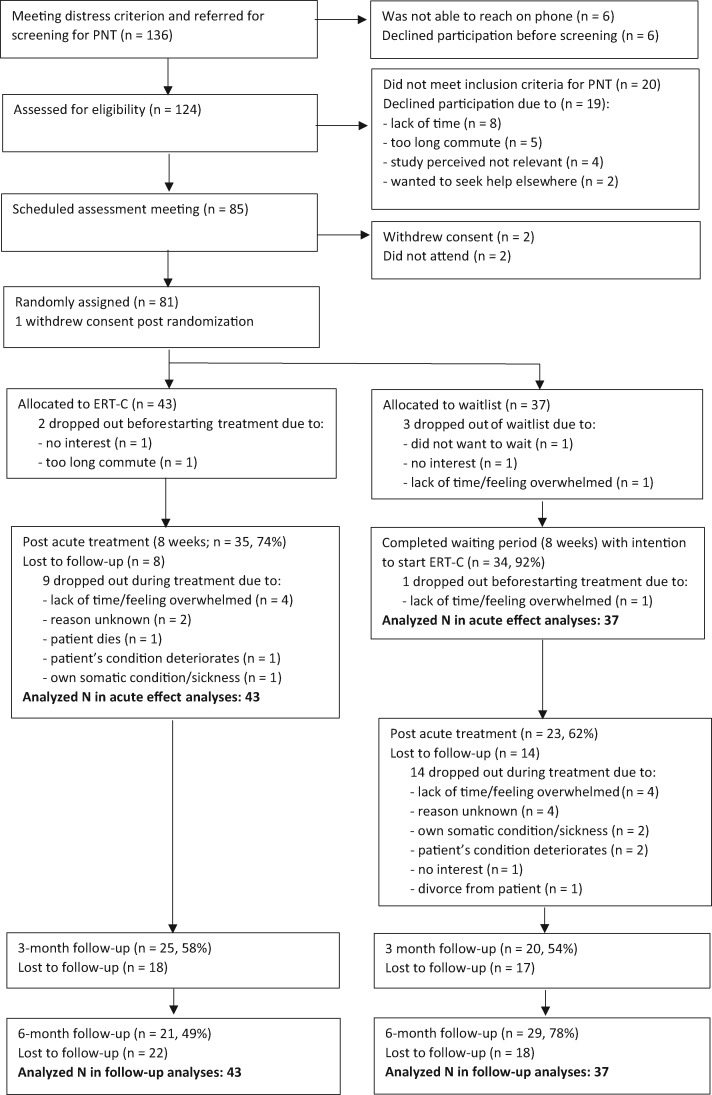
CONSORT flow diagram. ERT-C = Emotion Regulation Therapy adapted for caregivers; PNT = Preseverative negative thinking.

In total, 136 ICs were referred to further screening for perseverative negative thinking, of whom 124 were assessed for eligibility. Twenty ICs did not satisfy the inclusion and exclusion criteria, whereas 19 did but declined participation (see Figure 1 for reasons). Eighty-one ICs were randomly assigned, 43 to the ERT-C condition and 38 to the waitlist condition. One IC withdrew consent after randomization (see Figure 1). Of the 80 ICs, 95% (N* *=* *76) scored above the cutoff on worry (PSWQ) and 60% scored above the cutoff on rumination (N* *=* *48).

The mean age of the IC sample was 47.9 years (range = 18–69 years) and of the patients was 58.0 years (range = 29–73 years). Most ICs were female (75%) caring for male patients (73%). In 52 cases (65%), the patient cared for by the IC was enrolled, and in nine cases, there was more than one IC enrolled per patient. Most of the patients had stage IV cancer (58%) (see Table 1). Twenty-nine ICs (36%) dropped out, 23 during treatment (ERT-C; n* *=* *9). Treatment dropouts were statistically significantly older (mean* *=* *52.7 years) than completers (mean* *=* *45.3 years; *P* = .034) and worried less at pretreatment (*P* = .026). Dropouts were encouraged to complete scheduled questionnaires (46% did so). No dropouts were due to serious adverse events during treatment.

**Table 1.** Participant descriptives

Variable	ERT-C	Waitlist	Total
ICs, no.	43	37	80
Age, mean (SD), y	49.4 (15.2)	46.0 (16.1)	47.9 (15.6)
Women, %	74	75	75
Patients, no.	29	23	52
Age, mean (SD), y	60.2 (7.8)	55.0 (10.5)	58.0 (9.3)
Women, no. (%)	10 (35)	4 (17)	14 (27)
Cancer type, no. (%)			
Colon	7 (24)	6 (26)	13 (25)
Lung	4 (14)	7 (30)	11 (21)
Rectal	6 (21)	3 (13)	9 (17)
Ventricular	4 (14)	1 (4)	5 (10)
Pancreatic	1 (3)	0	1 (2)
Bile duct	1 (3)	0	1 (2)
Esophagus	2 (7)	0	2 (4)
Ovary	0	1 (4)	1 (2)
Uterus	0	1 (4)	1 (2)
Sarcoma	0	1 (4)	21 (2)
N/A*	4 (14)	3 (13)	7 (13)
Cancer stage, no. (%)	No. ()		
I	0 (0)	0 (0)	0 (0)
II	1 (4)	2 (9)	3 (6)
III	6 (20)	6 (26)	12 (21)
IV	18 (62)	12 (60)	30 (58)
N/A*	4 (14)	3 (13)	7 (13)
Primary treatment, no. (%)	No. ()		
Chemotherapy	15 (52)	12 (52)	27 (52)
Chemotherapy and surgery	7 (24)	5 (22)	12 (22)
Targeted biological treatment	2 (7)	1 (4)	3 (6)
Radiation and chemotherapy	1 (3)	2 (9)	3 (6)
N/A*	4 (14)	3 (13)	7 (13)

∗ Seven patients consented to complete questionnaires but did not consent to data extraction from their patient file. ERT-C = Emotion Regulation Therapy adapted for caregivers; IC = informal caregiver; N/A = not available.

### Acute Treatment Effects

#### Primary Outcomes for ICs

**Figure 2.**
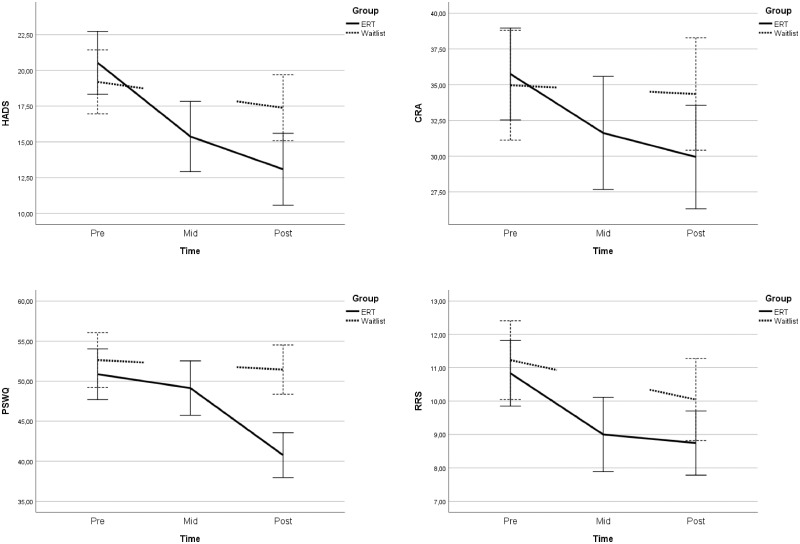
Effects on primary outcomes during acute treatment. Error bars depict 95% confidence interval. The Emotion Regulation Therapy adapted for caregivers group was assessed pre-, mid- (4 weeks), and post-treatment (8 weeks). The waitlist group was assessed pre- and post-treatment. CRA = Caregiver Reaction Assessment; HADS = Hospital Anxiety and Depression Scale; PSWQ = Penn State Worry Questionnaire; RRS = Ruminative Response Styles-Brooding subscale.

Compared with the waitlist condition, ICs receiving ERT-C experienced statistically significant reductions in psychological distress (*g*= 0.86, 95% confidence interval (CI) = 0.40 to 1.32, *P* < .001), worry (*g* = 0.96, 95% CI = 0.50 to 1.42, *P* < .001), and caregiver burden (*g* = 0.53, 95% CI = 0.10 to 1.99, *P* = .007) post-treatment, corresponding to medium to large effect sizes. No statistically significant effect was found for rumination (*g* = 0.24, 95% CI = −0.20 to 0.68, *P* = .220) (see Table 2; Figure 2).  

**Table 2.** Means (SDs) and interaction effects (group × time) of acute treatment

	ERT			Waitlist		Group × time		
Variable	Pre (IC n = 43)	Mid (IC n = 32)	Post (IC n = 35)	Pre (IC n = 37)	Post (IC n = 34)	F	*P*	*g* (CI)
Primary caregiver outcomes*								
HADS (n = 79)	20.5 (7.1)	15.4 (6.8)	13.1 (7.3)	19.2 (6.6)	17.4 (6.6)	19.9	<.001	0.86 (0.40 to 1.32)
PSWQ (n = 77)	50.9 (10.2)	49.1 (9.4)	40.8 (8.1)	52.6 (10.0)	51.4 (8.8)	23.6	<.001	0.96 (0.50 to 1.42)
CRA (n = 79)	35.7 (10.4)	31.6 (11.0)	29.9 (10.5)	35.0 (11.4)	34.3 (11.3)	7.9	.006	0.55 (0.10 to 1.00)
RRS-B (n = 77)	10.8 (3.2)	9.0 (3.1)	8.7 (2.8)	11.2 (3.4)	10.0 (3.5)	1.5	.220	0.24 (−0.20 to 0.68)
Secondary caregiver outcomes								
WHO-5 (n = 79)	9.2 (5.3)	12.5 (5.0)	15.5 (4.7)	10.2 (5.4)	12.3 (5.6)	15.9	<.001	0.79 (0.33 to 1.25)
PSQI (n = 78)	9.3 (3.1)	8.4 (3.7)	6.5 (2.6)	9.4 (3.6)	7.7 (3.9)	4.3	.043	0.51 (0.40 to 1.32)
Emotional closeness (n = 78)	−1.1 (2.8)	−1.8 (1.7)	−1.7 (1.5)	−1.7 (1.7)	−1.5 (1.4)	2.1	.151	0.28 (0.06 to 0.96)
CRP (n = 66)	2.6 (3.3)	—	1.7 (1.4)	2.5 (3.1)	2.8 (3.4)	0.3	.570	0.17 (−0.27 to 0.61)
IL-1β† (n = 10)	—	—	—	—	—	—	—	—
IL-6 (n = 70)	0.9 (0.8)	—	0.8 (0.5)	0.8 (0.5)	0.8 (0.4)	1.6	.205	0.35 (−0.09 to 0.79)
TNF-alpha (n = 73)	2.2 (0.4)	—	2.2 (0.5)	2.2 (0.5)	2.3 (0.6)	0.2	.686	0.11 (−0.33 to 0.55)
Model-related outcomes								
EQ (n = 79)	33.9 (5.8)	35.6 (6.1)	39.1 (6.2)	34.3 (9.4)	34.2 (8.0)	15.6	<.001	0.93 (0.47 to 1.39)
DERS (n = 79)	86.9 (20.7)	80.7 (22.5)	72.1 (20.8)	92.8 (24.2)	89.9 (24.2)	10.4	.002	0.77 (0.32 to 1.23)
FFMQ (n = 77)	125.5 (15.2)	131.0 (16.9)	139.0 (18.2)	118.9 (16.1)	120.0 (19.4)	19.2	<.001	0.92 (0.46 to 1.38)
ERQ-R (n = 79)	26.4 (5.7)	27.1 (5.4)	29.0 (6.1)	27.1 (6.9)	26.2 (6.1)	4.8	.031	0.43 (−0.02 to 0.88)
Patient outcomes	(PT n = 22)		(PT n = 16)	(PT n = 14)	(PT n = 11)			
EORCT-QLQ-C30 (n = 37)	53.4 (17.0)	—	67.2 (18.6)	57.2 (21.1)	54.5 (26.7)	6.1	.019	0.88 (0.18 to 1.58)
HADS (n = 36)	12.1 (6.3)	—	8.9 (5.0)	13.0 (13.8)	11.6 (8.4)	0.2	.672	0.14 (−0.53 to 0.81)
CRP (n = 36)	10.7 (12.3)	—	12.6 (26.2)	7.6 (10.9)	5.6 (4.4)	2.1	.162	0.61 (−0.08 to1.30)
IL-1β† (n = 10)	—	—	—	—	—	—	—	—
IL-6 (n = 38)	2.0 (1.5)	—	1.7 (1.1)	2.6 (3.7)	1.3 (0.7)	0.5	.487	0.30 (−0.37 to 0.97)
TNF-alpha (n = 38)	2.7 (0.8)	—	2.6 (0.5)	2.6 (0.9)	2.4 (0.7)	0.3	.581	0.24 (−0.43 to 0.91)

* Numbers following the outcome variable names refer to number of records completed pretreatment. CI = confidence interval; CRA = Caregiver Reaction Assessment; CRP = C-reactive protein; DERS = Difficulties with Emotion Regulation Scale; EORCT-QLQ = European Organization for Research and Treatment of Cancer Core Quality of Life Questionnaire; EQ = Experience Questionnaire; ERQ-R = Emotion Regulation Questionnaire-Reappraisal subscale; ERT = Emotion Regulation Therapy; FFMQ = Five Facet Mindfulness Questionnaire; HADS = Hospital Anxiety and Depression Scale; IC = informal caregiver; IL = interleukin; PSQI = Pittsburgh Sleep Quality Index; PSWQ = Penn State Worry Questionnaire; PT = patient; RRS-B = Ruminative Response Styles-Brooding subscale; TNF = tumor necrosis factor; WHO-5 = World Health Organization-5 Well-Being Index.

† Insufficient data (N* *=* *10).

#### Secondary and Model-Related Outcomes for ICs

Statistically significant changes in the expected direction were observed for all self-report secondary outcomes at post-treatment (all *P* values < .05), except for emotional closeness, with changes corresponding to medium to large effect sizes (*g* = 0.51–0.79). All emotion regulation model-related outcomes also improved (all *P* values < .05), corresponding to medium to large effect sizes (*g* = 0.43–0.93). No statistically significant changes in inflammatory outcomes were detected (all *P* values > .2) (see Table 2).

#### Patient Outcomes

Patients of ICs receiving ERT-C experienced, compared with patients in the waitlist condition, improved overall quality of life (*P* = .019, *g* = 0.88) at post-treatment, whereas no such statistically significant changes were observed for psychological distress (*g* = 0.14, *P* = .672) or inflammatory outcomes (all *P* values > .2).

### Treatment Effects at Follow-Up

When all ICs had received ERT-C, the two groups were combined in the evaluation of the long-term treatment effect (ie, through 6 months follow-up). ICs receiving ERT-C immediately or delayed experienced equivalently positive effects across the acute delivery of ERT-C, with the only exception being WHO-5, where ICs immediately receiving ERT-C experienced a statistically larger effect (*P <* .001; *g *=* *0.67).

**Figure 3.**
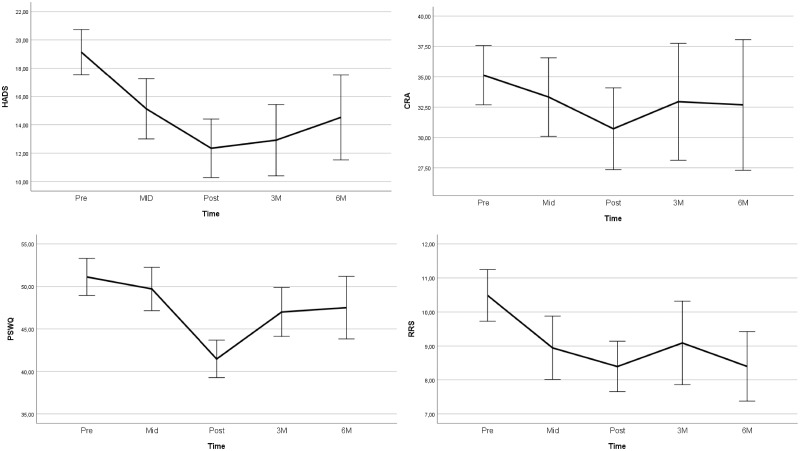
Effects on primary outcomes through the follow-up period. Error bars depict 95% confidence interval. The lines represent within-person change over time in the two groups combined from pre-, mid- (4 weeks), and post-treatment (8 weeks) to 3 months and 6 months follow-up. CRA = Caregiver Reaction Assessment; HADS = Hospital Anxiety and Depression Scale; PSWQ = Penn State Worry Questionnaire; RRS = Ruminative Response Styles-Brooding subscale.

Concerning primary outcomes, the obtained effects were of a medium magnitude (HADS, *g = *0.75; PSWQ, *g *=* *0.62), although a worsening was observed from post-treatment through the follow-up period for both psychological distress (HADS) and worry (PSWQ), as indicated by a quadratic effect of time. Effects obtained in terms of caregiver burden (CRA) were maintained. See Table 3 and Figure 3. Concerning secondary outcomes, effects were maintained through follow-up except for quality of life, which deteriorated during the follow-up period, although the overall effect was medium (WHO; *g *=* *0.71). The effect on patient quality of life obtained following acute treatment also decreased to a small magnitude (EORCT-QLQ-C30; *g *=* *0.34) and was statistically non-significant.

**Table 3.** Long-term treatment effects for the total sample having received Emotion Regulation Therapy adapted for caregivers presented as means (SDs)

	Pre	Mid	Post	3 months	6 months	Pretreatment to 6 mo follow-up *P*/effect size (g)∗
Outcomes	IC n = 80	IC n = 54	IC n = 58	IC n = 45	IC n = 45	
Primary caregiver outcomes (best fit of time†)						
HADS (quadratic)	19.1 (7.0)	15.1 (7.8)	12.3 (7.9)	12.9 (8.4)	14.5 (9.4)	<.001/0.75
PSWQ (quadratic)	51.1 (9.5)	49.1 (9.3)	41.5 (8.4)	47.0 (9.6)	47.5 (11.3)	<.001/0.62
CRA (loglinear)	35.1 (10.8)	33.3 (11.9)	30.7 (12.8)	32.9 (16.0)	32.7 (16.6)	.016/0.32
RRS (loglinear)	10.5 (3.3)	8.9 (3.4)	8.4 (2.8)	9.1 (4.1)	8.4 (3.2)	<.001/0.66
Secondary caregiver outcomes (best fit of time)						
WHO‡ (quadratic)	10.6 (5.6)	12.4 (5.8)	15.2 (5.5)	14.1 (5.9)	14.0 (5.8)	<.001/0.71
PSQI (loglinear)	8.6 (3.5)	7.6 (3.6)	6.1 (2.7)	7.1 (3.6)	6.9 (4.6)	<.001/0.53
Emotional closeness (log-linear)	−1.3 (2.3)	−1.8 (1.6)	−1.6 (1.5)	−1.3 (2.9)	−2.2 (2.7)	.287/0.14
Model-related outcomes						
EQ (loglinear)	34.1 (6.8)	35.5 (7.0)	39.4 (6.5)	39.6 (7.7)	38.7 (7.4)	<.001/1.00
DERS (loglinear)	88.3 (22.2)	83.8 (24.0)	72.6 (20.1)	70.7 (19.4)	70.4 (19.1)	<.001/1.34
FFMQ (loglinear)	123.2 (17.3)	127.4 (18.4)	136.1 (19.5)	139.2 (20.0)	136.8 (18.9)	<.001/1.19
ERQ-R (loglinear)	26.3 (5.9)	26.9 (6.6)	29.6 (6.0)	29.1 (7.1)	30.0 (5.6)	<.001/0.54
Patient outcomes (best fit of time)	PT (n = 36)		PT (n = 27)	PT (n = 27)	PT (n = 21)	
EORCT (quadratic)	53.8 (20.7)	—	63.9 (20.5)	65.4 (23.1)	59.9 (30.1)	.109/0.34
HADS (loglinear)	11.9 (7.0)	—	9.4 (6.0)	9.2 (6.9)	10.7 (8.1)	.274/0.24

∗ Effect sizes refer to estimations based on linear effects from pre-treatment through the follow-up period in order to compare end-point effects. CRA = Caregiver Reaction Assessment; DERS = Difficulties with Emotion Regulation Scale; EORCT = European Organization for Research and Treatment of Cancer Core Quality of Life Questionnaire; EQ = Experience Questionnaire; ERQ-R = Emotion Regulation Questionnaire-Reappraisal subscale; FFMQ = Five Facet Mindfulness Questionnaire; HADS = Hospital Anxiety and Depression Scale; IC = informal caregiver; PSQI = Pittsburgh Sleep Quality Index; PSWQ = Penn State Worry Questionnaire; PT = patient; RRS = Ruminative Response Styles-Brooding subscale; WHO-5 = World Health Organization-5 Well-Being Index.

† Best fit of time. Three functions of time were evaluated in order to determine the best fit of time, including a linear effect.

‡ Statistically significant difference between the immediate and delayed group during the acute treatment, favoring the immediate group (indicative of continued improvement), a log-linear effect (indicative of effect maintenance), and a quadratic effect (indicative of worsening). This evaluation was based on fit statistics, taking into account the number of parameters in the model.

## Discussion

ERT-C demonstrated positive effects on three of four primary outcomes corresponding to medium to large effects for psychological distress, worry, and caregiver burden, and exceeding the suggested cutoff for a minimally important difference (*d *=* *0.50) (51). Demonstrating such positive effects largely replicates findings from our open-label, single arm of ERT-C for distressed ICs (23) and is especially encouraging given that previous CBTs have failed to efficaciously treat IC distress (12). The finding concerning rumination was not statistically significant and goes counter to our open trial and may reflect that the present sample was considerably more characterized by worry than rumination. At baseline, our IC sample scored above the clinical cutoff for worry (PSWQ = 45) (52) with an overall mean of 51, indicative of pathological worry, and where 95% of ICs were included based on their worry score, only 60% scored above the cutoff on rumination. 

Concerning secondary outcomes, statistically significant effects of medium magnitudes were detected for both quality of life (WHO-5) and sleep quality (Pittsburgh Sleep Quality Index). Emotional closeness did not improve, and, finally, we were unable to detect any improvement on proinflammatory outcomes. The latter finding should be viewed in light of the small effect of psychotherapies on inflammatory markers detected in a recent meta-analysis (*g *=* *0.15) (27) and the detected small effect sizes in the present study (*g* range = 0.11–0.35). This study was thus not powered to detect such small effects.

Given the waitlist control design of the study, treatment durability effects reflect uncontrolled, within-person change. The encouraging effects detected following the acute treatment period were largely maintained through the 3- and 6-month follow-up. Although some outcomes deteriorated, as indicated by a quadratic effect of time, the endpoint effects, except two (CRA and emotional closeness), remained medium to large in magnitude (*g *=* *0.54–1.34). Deterioration of the effect could reflect progression of the cancer and the increased caring responsibilities. It could also be indicative of emotion regulation skills not yet being solidified and a need for booster sessions.

Beyond the positive outcomes for ICs, the cancer patients also evidenced improvements in terms of quality of life corresponding to a large effect (*g *=* *0.88) even though they received no therapy. Despite an attenuation of effect through follow-up, this finding is particularly encouraging because many patients are burdened with intense treatment programs. The increase in quality of life for cancer patients indicates a buffering effect of IC mental health on patient quality of life. 

Limitations of the present study include the relatively small number of included participants. Moreover, a majority of female IC participants were included and there was a 35% treatment dropout rate. This dropout rate falls within the range of attrition detected in previous trials for ICs (mean = 28%; range = 0–49 [53]), but it should be noted that the most frequent reason for dropping out was lack of time or feeling overwhelmed and that there was a selective dropout of older and less worried ICs, precluding meaningful comparisons of treatment gains between completers and noncompleters. In addition, although based on the intent-to-treat sample, the results obtained from the follow-up analyses are limited by a study completion rate of 63%. Second, employing a waitlist control group prevents conclusions about the specificity of ERT. In addition, the findings concerning the follow-up period are limited by the lack of a control group during this time. Third, too few observations were available for IL-1β to conduct the planned analyses (ie, below detection or fit curve range). Other inflammation results are limited by the suboptimal procedures of the current study with only two assessment time points. Future studies may consider assessing inflammatory activity in response to a contextually meaningful stressor rather than simple pre-post assessments (54). Fourth, more information on ICs choosing not to participate despite elevated levels of distress is needed to evaluate the generalizability of the current results and to develop treatment options with a strong appeal to this population. Finally, without a cost-effectiveness analysis, which was precluded due to our design where the controlled effect was limited to the initial 8 weeks, we cannot evaluate the detected benefits in light of their associated economic burden. An important future direction will be to conduct a trial of ERT-C against a well-equated comparator to evaluate efficacy as well as cost effectiveness. 

In conclusion, ICs receiving ERT-C experienced positive effects with regard to three out of four primary outcomes. Although some deterioration was detected from post-treatment through the follow-up period, effects on the combined sample remained of medium to large magnitudes. In addition, patients whose caregivers received ERT-C experienced a large increase in quality of life. Given that ICs are integral to the care provided to patients with cancer but often in need of care themselves, identifying ICs with elevated psychological distress and providing them with relevant psychotherapy appears an important element of comprehensive cancer care. Our encouraging findings warrant further research in an adequately powered, multi-center, phase III trial designed to determine net clinical benefits.

## Funding

This work was supported by the Danish Cancer Society (#R119-A7545;#R96-A6385).

## Notes

Affiliations of authors: Unit for Psychooncology and Health Psychology, Aarhus University and Aarhus University Hospital, Denmark (MSO, RZ); Department of Psychology, Teachers College, Columbia University, New York, NY (DSM); Department of Psychiatry & Behavioral Sciences, Memorial Sloan Kettering Cancer Center, New York, NY (AA); Department of Oncology, Aarhus University Hospital, Denmark (BW, HR); Department of Psychological Sciences, Kent State University, Kent, OH (DMF).

The authors declare no conflict of interest.
